# Transmembrane domain-mediated Lck association underlies bystander and costimulatory ICOS signaling

**DOI:** 10.1038/s41423-018-0183-z

**Published:** 2018-12-06

**Authors:** Zurong Wan, Xingxing Shao, Xingyu Ji, Lihui Dong, Jiacheng Wei, Zhuqing Xiong, Wanli Liu, Hai Qi

**Affiliations:** 1 Tsinghua-Peking Center for Life Sciences, Tsinghua University, 100084 Beijing, China; 2 Laboratory of Dynamic Immunobiology, Institute for Immunology, 100084 Beijing, China; 3 Department of Basic Medical Sciences, School of Medicine, 100084 Beijing, China; 4 School of Life Sciences, 100084 Beijing, China; 5 Beijing Key Lab for Immunological Research on Chronic Diseases, 100084 Beijing, China; 6 MOE Key Laboratory of Protein Sciences, Tsinghua University, 100084 Beijing, China

**Keywords:** Follicular T-helper cells, Signal transduction, Tumour immunology

## Abstract

The B7-family inducible costimulator (ICOS) activates phosphoinositide-3 kinase (PI3K) and augments calcium mobilization triggered by the T-cell receptor (TCR). We surprisingly found that the entire cytoplasmic domain of ICOS is dispensable for its costimulation of calcium mobilization. This costimulatory function relies on the unique transmembrane domain (TMD) of ICOS, which promotes association with the tyrosine kinase Lck. TMD-enabled Lck association is also required for p85 recruitment to ICOS and subsequent PI3K activation, and Lck underlies both the bystander and costimulatory signaling activity of ICOS. TMD-replaced ICOS, even with an intact cytoplasmic domain, fails to support T_FH_ development or GC formation in vivo. When transplanted onto a chimeric antigen receptor (CAR), the ICOS TMD enhances interactions between T cells and antigen-presenting target cells. Therefore, by revealing an unexpected function of the ICOS TMD, our study offers a new perspective for the understanding and potential application of costimulation biology.

## Introduction

The inducible costimulator (ICOS) molecule is a CD28-family costimulatory receptor that is upregulated by T cells following antigen stimulation.^1,2^ While ICOS is expressed by all recently activated T cells in vivo, its expression level is particularly high on follicular T-helper (T_FH_) cells, the CD4^+^ T-cell subset that specializes in promoting the germinal center response and humoral immunity.^3^ It is well established that ICOS is crucial for the development and function of T_FH_ cells and is thus required for the germinal center reaction and affinity-matured antibody responses.^4–10^ At the cellular level, ICOS functions in two context-dependent modes in vivo.^11^ In the bystander mode, ICOS on activated T cells is engaged by follicular B cells that do not present antigen but constitutively express ICOS ligand (ICOSL). This bystander engagement of ICOS does not involve concomitant TCR triggering but enhances PI3K activation in the T cells, which promotes T-cell motility and follicular recruitment.^12^ In the costimulatory mode, on the other hand, ICOS promotes TCR-dependent CXCR5 upregulation during priming interactions with antigen-presenting dendritic cells.^9,13,14^ During antigen-specific interactions between T_FH_ cells and cognate B cells in the follicle and the germinal center, ICOS enhances TCR-dependent calcium mobilization, which drives efficient CD40L externalization from vesicular stores in T cells and subsequent delivery to interacting B cells.^15^

At the molecular level, however, it is not yet clear how ICOS signals to accomplish these cellular functions. Previous reports have established that when tyrosine-phosphorylated, the conserved YMFM motif in the ICOS cytoplasmic domain recruits the regulatory subunit p85 of phosphoinositide-3 kinase (PI3K) and activates the PI3K pathway.^4,16,17^ Importantly, constitutive p85 binding to ICOS can be detected in recently activated T cells without overt TCR stimulation or ICOS ligation, and ICOS ligation further enhances p85 binding and PI3K activation.^12,16,18^ Given that no kinase is known to associate with ICOS, it is not clear how ICOS can be constitutively phosphorylated and bound with p85 and how this recruitment is enhanced after its own crosslinking.

In contrast to PI3K activation, ICOS ligation alone does not trigger calcium flux, and ICOS-enhanced calcium mobilization strictly depends on concomitant TCR stimulation.^15^ ICOS costimulation of TCR-triggered calcium flux was originally thought to be a secondary effect of ICOS-driven PI3K activation.^17,19^ However, upon analyzing a knockin mouse strain carrying the ICOS^Y181F^ allele, which renders the ICOS YMFM motif phosphorylation-incompetent, Gigoux and colleagues demonstrated that the ability to activate PI3K is not required for ICOS to costimulate calcium mobilization.^18^ What actually underlies ICOS-mediated calcium costimulation therefore remains unclear.

In the current study, we surprisingly found that the entire cytoplasmic domain of ICOS is not necessary for its costimulation of TCR-triggered calcium mobilization. Instead, the unique transmembrane domain (TMD) of ICOS promotes its weak but constitutive association with the tyrosine kinase Lck and thereby augments proximal TCR signaling to enhance calcium mobilization. Furthermore, the unique ICOS TMD is also required for p85 recruitment and normal PI3K activation following ICOS ligation, a process that also depends on Lck. Therefore, by associating with Lck in a TMD-dependent manner, ICOS can constitutively signal through PI3K-dependent pathways without ligand interactions, enhance its Lck association and consequent PI3K activation upon receptor ligation and, when coligated together with the antigen receptor complex, augment the proximal signaling output by providing an extra pool of Lck.

## Materials and methods

### Mice

B6 (Jax 664), *Icos*^-/-^ (Jax 4859), OVA_323-339_-specific TCR transgenic OT-II (Jax 4194), and HEL-specific Ig-transgenic MD4 (Jax 2595) mice were originally from the Jackson Laboratory. All mice were maintained under specific pathogen-free conditions and used in accordance with governmental and institutional guidelines for animal welfare.

### ICOS-expressing Jurkat cells

The parental Jurkat cell line was a kind gift from Dr. Chenqi Xu. Lentivirus was produced in 293T cells cotransfected with the psPAX2 and pMD2.G packaging plasmids together with a pHAGE vector that expresses wild-type ICOS or various mutant ICOS molecules and GFP. For lentiviral transduction, 5 × 10^5^ Jurkat cells in the logarithmic growth phase were spin-infected at 1500 × *g* with appropriate viral supernatants in the presence of 4 μg/ml polybrene (Sigma-Aldrich) for 2 h at 32 °C. Transduced Jurkat cells were subsequently propagated and used either as bulk culture or after further sorting of GFP^+^ Jurkat cells by flow cytometry to >99% purity.

### Lck-deficient Jurkat cells

A subline of Jurkat cells that performs well in electroporation, a kind gift from Dr. Yun-Cai Liu, was used to create Lck-deficient Jurkat cells. The cells were electroporated with MSCV-based GFP-tagged vectors that express Cas9 and Lck-targeting guide RNAs. Single GFP^+^ Jurkat cells were cloned by sorting and were genotyped by PCR. Two Jurkat clones (clone 1 and clone 16) that lacked the guide RNA-targeted fragment from exon 2 to exon 9 were selected for subsequent experiments after validation by Western blotting (see Figure S5 for details). For experiments involving ICOS stimulation or immunoprecipitation in the absence of Lck, these Lck-deficient Jurkat cells and corresponding wild-type control cells were further transduced with ICOS-expressing constructs as described above.

### Calcium mobilization assay

Pilot experiments were conducted to determine the anti-CD3 antibody concentration optimal for observation of the ICOS-mediated costimulation of calcium mobilization in primary mouse T cells and in the Jurkat T cell line.

Regarding the primary mouse T cells, CD4^+^ T lymphocytes were isolated by CD4 Microbeads (Miltenyi Biotec) from *Icos*^-/-^ mice and activated with plate-bound anti-CD3 and anti-CD28 antibodies (Bio X Cell) in complete RPMI 1640 media. Retroviral transduction of these T cells with desired ICOS-expressing constructs or an empty vector was performed as previously described.^12^ Transduced T cells were typically cultured for 4 days before use. To assay calcium flux, cells (10^7^ per ml) were stained with 2 mM Indo-1 (Invitrogen) in RPMI medium without serum at 37 °C for 30 min, washed twice with ice-cold RPMI containing 1% serum, and then incubated in ice-cold RPMI containing 1% serum with biotinylated anti-CD3 (145-2C11, eBioscience, 2.5 μg/ml) and/or biotinylated anti-ICOS antibodies (C398.4A, BioLegend, 5 μg/ml) at a final cell density of 4 × 10^6^ per ml for 1 h. The cells were washed twice with RPMI containing 1% serum and brought to 37 °C to read baseline Indo-1 fluorescence signals by flow cytometry for 1 min, followed by an additional 4-min recording after streptavidin was added to the cell suspension at a final concentration of 50 μg/ml. To minimize the effects of tube-to-tube variation, GFP^+^ and GFP^−^ cells in the same tube were compared to determine ICOS-mediated costimulation.

Jurkat cells (5 × 10^6^ per ml) were stained with 2 mM Indo-1 (Invitrogen) in HBSS at 37 °C for 30 min, washed once, and then suspended in Ringer’s solution containing 2 mM Ca^2+^. Cells (2 × 10^6^ per ml) were then incubated with biotinylated anti-CD3 (OKT3, BioLegend, 0.2 μg/ml) and biotinylated anti-ICOS antibodies (C398.4A, BioLegend, 2 μg/ml) at room temperature for 1 min. Indo-1 fluorescence was then measured for 1 min, followed by an additional 4-min recording after streptavidin was added to the cell suspension at a final concentration of 50 μg/ml. For certain experiments, Indo-1-loaded Jurkat cells were suspended in calcium-free Ringer’s solution to incubate with biotinylated anti-CD3 and biotinylated anti-ICOS antibodies, baseline Indo-1 fluorescence was read for 1 min, streptavidin was added to a final concentration of 50 μg/ml to monitor Indo-1 fluorescence for another 2 min, and Ca^2+^ was added to a final concentration of 2 mM for an additional 3-min recording. Where applicable, GFP^−^ Jurkat cells were spiked into individual tubes containing different GFP^+^ transduced Jurkat cells to minimize the potential impacts of tube-to-tube variation on data interpretation, and each experiment was always conducted with its own GFP^-^ or empty vector to control for batch variations of the stimulating reagents.

### Immunoprecipitation and Western blotting

To probe p85 in ICOS immunoprecipitates after ICOS stimulation, 2 × 10^7^ Jurkat cells were suspended in 100 μl of serum-free RPMI, mixed with 100 μl of prewarmed serum-free RPMI containing the anti-ICOS antibody (C398.4A, 10 μg/ml, BioLegend) and a goat anti-hamster secondary antibody (20 μg/ml, Invitrogen), and then incubated at 37 °C for 1 min. The cells were then washed with 10 ml of precooled PBS and lysed on ice for 10 min with 500 μl of RIPA buffer that contained 50 mM Tris-Cl pH = 7.4, 0.25% sodium deoxycholate, 150 mM NaCl, 10 mM EDTA, 1% protease inhibitor cocktail (Bimake), and 2% phosphatase inhibitor (Bimake). After centrifugation at 21,000 × *g* for 10 min to remove debris, 3% of the lysate was taken as input, and the remaining lysate was incubated with 20 μl of Protein A/G PLUS agarose beads (Santa Cruz) overnight at 4 °C. The beads were then washed five times with ice-cold RIPA buffer, and the immunoprecipitates were eluted by adding 40 μl of SDS-containing sample buffer and boiling at 100 °C for 10 min. To prepare immunoprecipitates from unstimulated control cells, Jurkat cells were kept on ice for 10 min before the addition of 100 μl of precooled serum-free RPMI containing the anti-ICOS antibody (C398.4A, BioLegend, 10 μg/ml) and a secondary antibody (goat anti-hamster, 20 μg/ml, Invitrogen) and further incubation on ice for 5 min. These cells were then washed, lysed, and subjected to immunoprecipitation in parallel with the stimulated cells.

To probe mCherry-tagged Lck in ICOS immunoprecipitates, 3 × 10^7^ Jurkat cells were suspended in 700 μl of precooled serum-free RPMI containing the anti-ICOS antibody (C398.4A, 10 μg/ml, BioLegend) and then incubated on ice for 30 min. The cells were then washed with 10 ml of precooled PBS and lysed on ice for 10 min with 700 μl of Brij97 buffer containing 20 mM HEPES (pH = 7.4), 150 mM NaCl, 1% Brij97, 1% protease inhibitor cocktail (Bimake), and 2% phosphatase inhibitor (Bimake). After centrifugation at 21,000×*g* for 10 min to remove debris, 3% of the lysate was taken as input, and the remaining lysate was incubated with 30 μl of Protein A/G PLUS agarose beads (Santa Cruz) overnight at 4 °C. The beads were then washed three times with ice-cold Brij97 buffer and once with detergent-free buffer containing 20 mM HEPES (pH = 7.4) and 150 mM NaCl, and the immunoprecipitates were eluted by adding 40 μl of SDS-containing sample buffer and boiling at 100 °C for 10 min.

To probe phospho-Akt, 3 × 10^5^ Jurkat cells were washed twice with PBS, suspended in 50 μl of serum-free RPMI, and further mixed with 50 μl of prewarmed antibody mixture containing the anti-ICOS antibody (C398.4A, 10 μg/ml, BioLegend) and a goat anti-hamster secondary antibody (20 μg/ml, Invitrogen). The cells were incubated at 37 °C for the indicated periods. The reactions were terminated by adding 100 μl of 2× TNE containing 0.1 M Tris-Cl (pH = 8.0), 4 mM EDTA, 2% Triton X-100, 2% protease inhibitor cocktail (Bimake), and 4% phosphatase inhibitor (Bimake) and incubating on ice for 10 min. The lysates were cleared by centrifugation at 21,000×*g* for 10 min at 4 °C.

To probe phospho-ZAP70 and phospho-PLCγ, 10^6^ Jurkat cells were washed twice with PBS and suspended in 25 μl of serum-free RPMI. The cell suspension was mixed with 25 μl of prewarmed antibody cocktail containing biotinylated anti-CD3 (OKT3, 0.4 μg/ml, BioLegend) and/or biotinylated anti-ICOS (C398.4A, 4 μg/ml, BioLegend) and incubated at 37 °C for 1 min. The cell suspension was then further mixed with  50μl of prewarmed streptavidin solution (100 μg/ml) and incubated for 30 s. The reaction was terminated by adding 100 μl of 2× TNE containing 0.1 M Tris-Cl (pH = 8.0), 4 mM EDTA, 2% Triton-100, 2% protease inhibitor cocktail (Bimake), and 4% phosphatase inhibitor (Bimake) and incubating on ice for 10 min. The lysates were cleared by centrifugation at 21,000×*g* for 10 min at 4 °C.

The antibodies used for Western blotting included anti-p85 (Cell Signaling Technology), goat anti-ICOS (R&D), anti-Akt (Cell Signaling Technology), anti-phospho-Akt (Ser473) (D9E, Cell Signaling Technology), anti-phospho-Akt (Thr308) (Cell Signaling Technology), anti-β-actin (EASYBIO), anti-phospho-ZAP-70 (Tyr309)/Syk (Tyr352) (Cell Signaling Technology), anti-ZAP-70 (99F2) rabbit mAb (Cell Signaling Technology), anti-phospho-PLCγ (Tyr783) (Cell Signaling Technology), anti-PLCγ1 (DH910) XP rabbit mAb (Cell Signaling Technology), anti-Lck (3A5, Santa Cruz), anti-Fyn (FYN-59, Santa Cruz), goat anti-rabbit IgG-HRP (Easybio), goat anti-mouse IgG-HRP (Easybio), and donkey anti-goat IgG-HRP (Santa Cruz). The anti-mouse ICOS antibody used for Western blotting was a polyclonal antibody raised against the mouse ICOS extracellular domain from R&D (Cat# AF168). Due to glycosylation, ICOS is detected by this reagent as a smeared band between 34 and 43 kD and an additional band of approximately 26 kD when cells are lysed with NP-40 or as two to three bands of approximately 34 kD when lysed with Brij97.

### BioID

The basic method was previously developed,^20^ and the BirA*-coding vector was a kind gift of Dr. R. J. O’Sullivan. Jurkat cells transduced with BirA*-fused ICOS or ICOS-CD44TM were cultured in complete RPMI media supplemented with 50 μM biotin for 24 h. Cells (5 × 10^7^ per group) were washed with ice-cold PBS three times to remove free biotin before being lysed with 2 ml of RIPA buffer. The samples were kept on ice for 10 min prior to centrifugation at 21,000×*g* for 10 min to remove debris, and 3% of the lysate was taken from each sample as input prior to streptavidin pull-down. The remaining lysate of each sample was mixed with 50 μl of Dynabeads M-280 Streptavidin (Invitrogen) and incubated on a rotator at room temperature for 1 h. The beads were then washed with PBS containing 0.1% BSA 5 times, and the precipitates were eluted by adding 40 μl of SDS-containing sample buffer and boiling at 100 °C for 10 min.

### Assay of ICOS-Lck colocalization by TIRF microscopy

Jurkat T cells were placed on planar lipid bilayers (PLBs) that presented anti-ICOS antibodies. The PLBs were prepared as previously described.^21^ Briefly, biotin liposomes were prepared by sonication of 1,2-dioleoyl-sn-glycero-3-phosphocholine and 1,2-dioleoyl-sn-glycero-3-phosphoethanolamine-cap-biotin (Avanti Polar Lipids, Inc.) in a 25:1 molar ratio in PBS at a lipid concentration of 5 mM. Lab-Tek chambers (Thermo Fisher Scientific) were prepared with nanostrip-washed coverslips. The coverslips were incubated with 0.1 mM biotin liposomes in PBS for 30 min at 37 °C. After washing, the PLBs were incubated with 50 nM streptavidin for 30 min and then washed to remove excess streptavidin. The streptavidin-containing PLBs were further incubated for 30 min with 2 μg/ml biotinylated anti-ICOS antibody (C398.4A), blocked with 1% casein in PBS at 37 °C for 30 min and washed extensively with PBS before use. To evaluate ICOS-Lck colocalization, Jurkat T cells that expressed EGFP-tagged ICOS or ICOS-CD44TM and mCherry-tagged Lck were placed and incubated at 37 °C on the anti-ICOS antibody-incorporated PLBs for 10 min. The cells were then fixed with 1% paraformaldehyde for 30 min at 37 °C. The TIRF plane of individual cells was imaged using an Olympus IX-81 microscope equipped with a TIRF port, ANDOR iXon + DU-897D EMCCD camera, and Olympus 100× TIRF lens (1.49 NA oil). EGFP and mCherry were illuminated with 488-nm and 561-nm lasers, respectively (Coherent). Each imaged cell was analyzed using the colocalization function of Imaris (Bitplane), which took into account the point spread function of the imaging system, automatically calculated the colocalization, and yielded the Pearson’s correlation coefficient between the EGFP-ICOS and mCherry-Lck fluorescence for each cell.

### Functional rescue of *Icos*^-/-^ T cells with ICOS variants in vivo

Retrovirus was produced as previously described^12^ by transfecting Plat-E cells with an MSCV-based vector that expresses wild-type ICOS or mutant ICOS molecules together with IRES-GFP. *Icos*^-/-^ OT-II CD4 T lymphocytes were isolated from spleens with CD4 Microbeads (Miltenyi Biotec) and activated with plate-bound anti-CD3 and anti-CD28 antibodies (Bio X Cell). Retroviral transduction of T lymphocytes was performed as previously described.^12^ The transduced cells were sorted based on GFP expression levels. Equivalent surface ICOS levels among different groups were confirmed by surface staining of ICOS. To test the ICOS variants for CXCR5 upregulation, 2 × 10^7^ sorted T cells were adoptively transferred into B6 recipients and parked for 3 days before the recipients were immunized with 50 μg of OVA protein in alum together with 5 μg of LPS. CXCR5 expression on these T cells was assayed by flow cytometry 1 day after immunization. To test the ICOS variants for GC formation, 5 × 10^5^ sorted T cells were cotransferred together with 5 × 10^5^ MD4 B cells into B6 recipients, which were immunized subcutaneously with 130 μg of HEL-OVA conjugate antigen^22^ in alum supplemented with 1 μg of LPS 2 days later. The frequencies of MD4 GC B cells and OT-II T_FH_ cells were measured by flow cytometry 5 days after immunization.

### T-B conjugation assay for ICOS TM-modified CAR

Jurkat cells (10^4^ per reaction) expressing standard CD19-reactive BBz CAR that contained CD8TM as the transmembrane domain (a kind gift from Dr. Chenqi Xu) or the ICOSTM-swapped version (ICOSTM-BBz CAR) were mixed with 10^5^ TAMARA-labeled Farage B cells, centrifuged in a U-bottomed 96-well plate at 500×*g* for 3 min, and then incubated at 37 °C for 0.5 h. After vortexing for 40 s, the frequencies of conjugates were enumerated by flow cytometry as previously described.^23^ The Jurkat T cells were identified by GFP fluorescence provided by the CAR-expressing construct. Three technical replicates were assayed for each condition in every independent experiment.

## Results

### The cytoplasmic domain of ICOS is not required for its costimulatory function

To understand how ICOS may costimulate TCR-triggered calcium mobilization, we constructed a series of ICOS mutants, including a cytoplasmic domain-truncated version called ICOS-NT (Figure S1), and tested whether these mutant molecules could enhance TCR-triggered calcium mobilization when retrovirally transduced into primary *Icos*^-/-^ mouse T cells. As expected from previous findings by Gigoux and colleagues,^18^ the Y181F mutation did not affect surface ICOS expression (Figure S2A) and did not reduce calcium mobilization (Figure S2B). Unexpectedly, however, ICOS-NT was also able to costimulate calcium mobilization (Figure S2B).

The ICOS cytoplasmic domain is clearly indispensable for mediating the PI3K activation that underlies follicular recruitment and the maintenance of helper T cells during a GC response in vivo.^10–12,18^ However, ICOS plays a role in promoting CXCR5 upregulation within 24 h after antigen activation in vivo.^13^ To test whether ICOS-NT can be functional in vivo, *Icos*^-/-^ OT-II T cells transduced with ICOS or ICOS-NT were transferred into and activated in recipient mice following ovalbumin immunization. As shown in Figures S2C-E, control *Icos*^-/-^ OT-II T cells were not able to upregulate CXCR5 as efficiently as cells complemented with full-length ICOS, consistent with previous observations,^13^ but the ICOS-NT clearly rescued the defect. Therefore, ICOS-NT can functionally signal in vivo, and the striking implication is that ICOS-mediated calcium costimulation does not rely on the cytoplasmic domain.

**Fig. 1:**
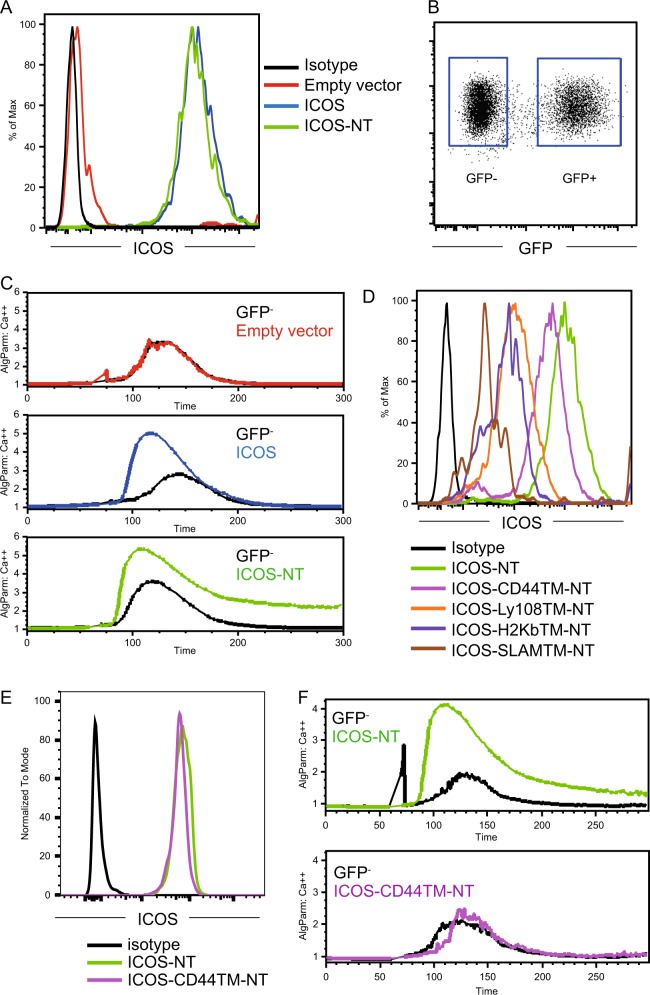
The ICOS transmembrane domain, but not the cytoplasmic domain, mediates the costimulation of calcium mobilization. **a** Surface ICOS staining of Jurkat cells lentivirally transduced with an empty vector or vectors expressing full-length ICOS or ICOS-NT. **b** Example of gating GFP^+^ stably transduced Jurkat cells and GFP^−^ spike-in nontransduced control Jurkat cells for the calcium mobilization assay. **c** Calcium mobilization in Jurkat cells of the indicated types and the respective internal GFP^-^ control cells after stimulation with biotinylated anti-CD3 (0.2 μg/ml) and anti-ICOS (2 μg/ml) antibodies coligated by streptavidin. The data represent the results of three independent experiments. **d** Surface ICOS staining of Jurkat cells lentivirally transduced with vectors expressing mutant ICOS molecules of the indicated types. **e** Surface ICOS staining after normalization of expression between ICOS-NT and ICOS-CD44TM-NT. **f** Calcium mobilization in Jurkat cells of the indicated types conducted as for **c**. The data represent the results of three independent experiments

ICOS-mediated functions are extremely sensitive to ICOS expression levels on the plasma membrane, and a mere 2-fold reduction may lead to functional compromise. To probe ICOS signaling mechanisms, therefore, it was important to obtain cells expressing ICOS and its variants at uniform and comparable levels on the cell surface. This was difficult using primary T cells. We therefore constructed stable ICOS-expressing Jurkat cell lines by lentiviral transduction. Endogenous ICOS expression on Jurkat cells is minimal (Fig. 1a, “Empty vector”). Importantly, as predicted, at a similar level of surface expression, ICOS-NT exhibited an ability to enhance calcium flux triggered by TCR stimulation comparable to that of full-length ICOS (Fig. 1b, c).

Using Jurkat cells, Leconte and colleagues recently suggested a membrane-proximal KKKY motif in the ICOS cytoplasmic tail to be important for ICOS-mediated calcium costimulation, although surface expression of KKKY-mutated ICOS was severely compromised,^24^ consistent with the fact that membrane-proximal polybasic residues promote efficient membrane-bound trafficking of transmembrane proteins.^25–28^ Those authors attempted to control for varied expression levels by simply applying a universal ICOS-expression gate during their calcium flux assays, even though gated cells still had overtly different levels of ICOS on the cell surface, precluding rigorous interpretations. Instead, we stringently normalized ICOS surface expression by sorting and subculturing Jurkat cells and found that ICOS-Y170F mutants, reported by Leconte et al. to impair calcium costimulation, were fully competent in costimulating calcium mobilization (Figures S3A-B).

Taken together, our findings rule out the possibility that a yet-to-be-identified signaling motif in the ICOS cytoplasmic domain is responsible for calcium costimulation. An intriguing possibility is that the ICOS transmembrane domain (TMD) might somehow signal into the cell.

### The ICOS transmembrane domain mediates the costimulation of calcium mobilization

To determine the potential role of the ICOS TMD, we constructed a series of hybrid ICOS-NT molecules that contained no cytoplasmic domain but rather contained the original ICOS extracellular domain anchored by different TMDs taken from other transmembrane protein molecules. Whereas the human ICOS TMD permitted similar levels of surface expression and supported costimulation of calcium mobilization with a high efficiency (Figures S4A-B), TMDs from H2K^b^, SLAM, Ly108, and CD44 all led to compromised surface ICOS expression (Fig. 1d). By cell sorting and subculturing, it was possible to derive ICOS-CD44TM-NT and ICOS-NT Jurkat cells that expressed comparable levels of ICOS on the cell surface (Fig. 1e). After normalization of surface expression, we found that coligation of CD3 and ICOS-NT led to significant calcium mobilization, but the calcium flux was much weakened in ICOS-CD44TM-NT Jurkat cells (Fig. 1f). These data indicate that the ICOS TMD is required for the costimulation of calcium signaling by ICOS.

### The ICOS transmembrane domain mediates costimulation by enhancing the proximal TCR signaling cascade

**Fig. 2:**
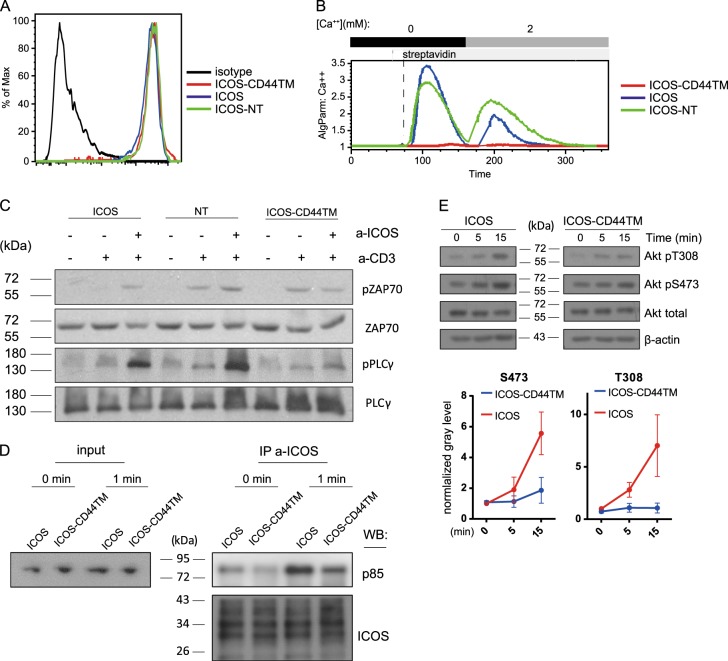
The ICOS transmembrane domain mediates calcium costimulation and is required for p85 recruitment. **a** Surface ICOS staining of Jurkat cells stably expressing ICOS, ICOS-CD44TM, or ICOS-NT molecules. **b** Calcium mobilization in Jurkat cells expressing ICOS molecules of the indicated types after stimulation with biotinylated anti-CD3 (0.2 μg/ml) and anti-ICOS (2 μg/ml) antibodies coligated by streptavidin. The data represent the results of three independent experiments. **c** Immunoblotting of phospho-ZAP70 and phospho-PLCγ in Jurkat cells expressing the indicated ICOS molecules 30 s after stimulation with anti-CD3 (0.2 μg/ml) and anti-ICOS (2 μg/ml) antibodies coligated by streptavidin. The data represent the results of three independent experiments. **d** Input (3% total lysates) or anti-ICOS immunoprecipitates of the indicated Jurkat cells before or 1 minute after ICOS crosslinking stimulation were probed by Western blotting for p85 and ICOS. **e** Immunoblotting of the indicated molecules in ICOS or ICOS-CD44TM Jurkat cells following anti-ICOS crosslinking stimulation for the indicated periods of time. Left, representative pAkt patterns; right, normalized pAkt levels of three independent experiments

To test whether the effect of ICOS costimulation impinges on ER calcium release or only calcium release-triggered calcium entry, we stimulated ICOS, ICOS-NT, and ICOS-CD44TM (Figure S1) Jurkat cells first in the absence and then in the presence of extracellular calcium. ICOS-CD44TM cells exhibited a dramatic defect in ER calcium release, even though surface expression levels of ICOS were comparable among all the cells (Fig. 2a, b). Furthermore, coligation of CD3 with ICOS or ICOS-NT, but not with ICOS-CD44TM, heightened ZAP70 and PLC-γ phosphorylation compared with anti-CD3 stimulation alone (Fig. 2c). These results demonstrate that ICOS costimulation of calcium mobilization indeed involves enhancement of proximal TCR signaling events, which primarily involve a phosphorylation cascade. An intriguing possibility is that the ICOS TMD may be directly or indirectly associated with a kinase. Because some ICOS molecules are constitutively phosphorylated and bound by p85 without TCR stimulation or ligand engagement in previously activated T cells, a putative ICOS TMD-associated kinase would explain how ICOS can mediate phosphorylation-dependent PI3K activation.

### The ICOS transmembrane domain is required for p85 recruitment and PI3K activation

We tested the prediction that the ICOS TMD is important for phosphorylation-dependent p85 recruitment to the ICOS cytoplasmic domain by examining p85 binding to ICOS before and after ICOS crosslinking. As shown in Fig. 2d, ICOS was constitutively bound by a certain level of p85, consistent with previous observations in recently activated primary T cells.^16,18^ On the other hand, p85 binding to ICOS-CD44TM molecules was markedly reduced, and this reduction was not corrected even after ICOS ligation. Consistent with this finding, enhanced Akt phosphorylation at T308 and S473 following ICOS ligation was significantly subdued when the CD44 transmembrane domain was used (Fig. 2e). These data demonstrate that the ICOS TMD is indeed important for phosphorylation-dependent p85 recruitment and PI3K activation under the steady-state and upon ICOS ligation, supporting the possibility that the ICOS TMD is associated with a kinase.

### Lck is required for p85 recruitment to ICOS and for ICOS costimulation

**Fig. 3:**
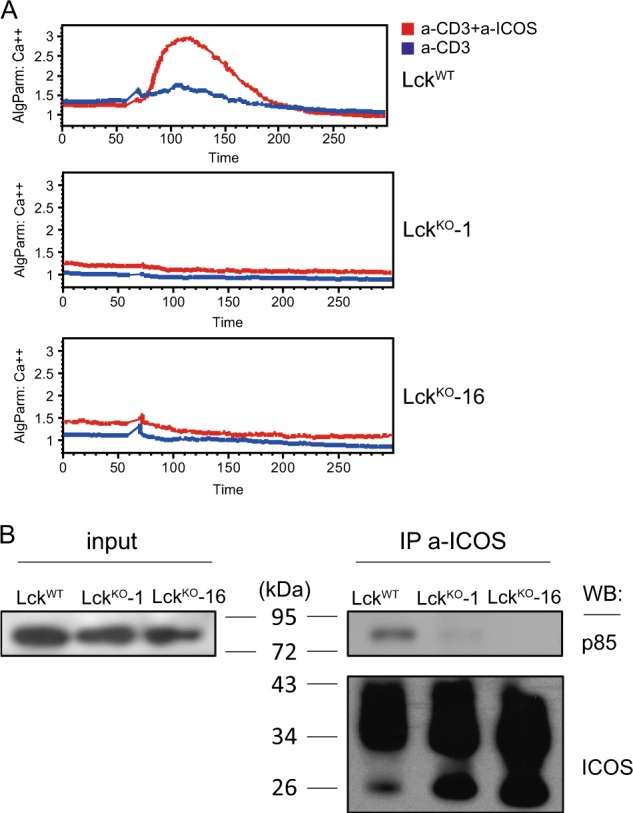
Lck is required for p85 recruitment to ICOS and for ICOS-mediated calcium costimulation. **a** Calcium mobilization by wild-type (WT) and *LCK*-knockout (KO) clone 1 and clone 16 Jurkat cells stably expressing wild-type ICOS after stimulation with anti-CD3 alone or anti-CD3 (0.2 μg/ml) and anti-ICOS (2 μg/ml) antibodies coligated by streptavidin. The data represent the results of three independent experiments. **b** Input (3% total lysates) or anti-ICOS immunoprecipitates of the indicated wild-type and Lck-deficient Jurkat cells were immunoblotted for p85 and ICOS. The data represent the results of three independent experiments

Lck is the primary upstream kinase that phosphorylates CD3 ITAMs and ZAP70 (ref. ^29^) and can be localized to the plasma membrane following myristylation and palmitylation^30^ To test whether Lck is required for ICOS functions, we used CRISPR technology to generate two independent clones of Lck-deficient Jurkat cells (Figure S5). After lentiviral transduction of ICOS, the two Lck-deficient clones were subjected to CD3 and ICOS coligation or CD3 stimulation alone. As shown in Fig. 3a, coligation failed to generate more calcium flux than anti-CD3 stimulation alone. Importantly, although the amount of ICOS that could be immunoprecipitated from LCK-deficient cells was consistently higher than that from LCK-sufficient cells, presumably due to the changed phosphorylation status of the molecule, p85 became essentially undetectable in the precipitates of the former (Fig. 3b), indicating that constitutive p85-ICOS binding depends on Lck. Therefore, Lck is functionally required for both ICOS-mediated calcium costimulation and constitutive PI3K activation.

### Transmembrane domain-dependent ICOS-Lck association

**Fig. 4:**
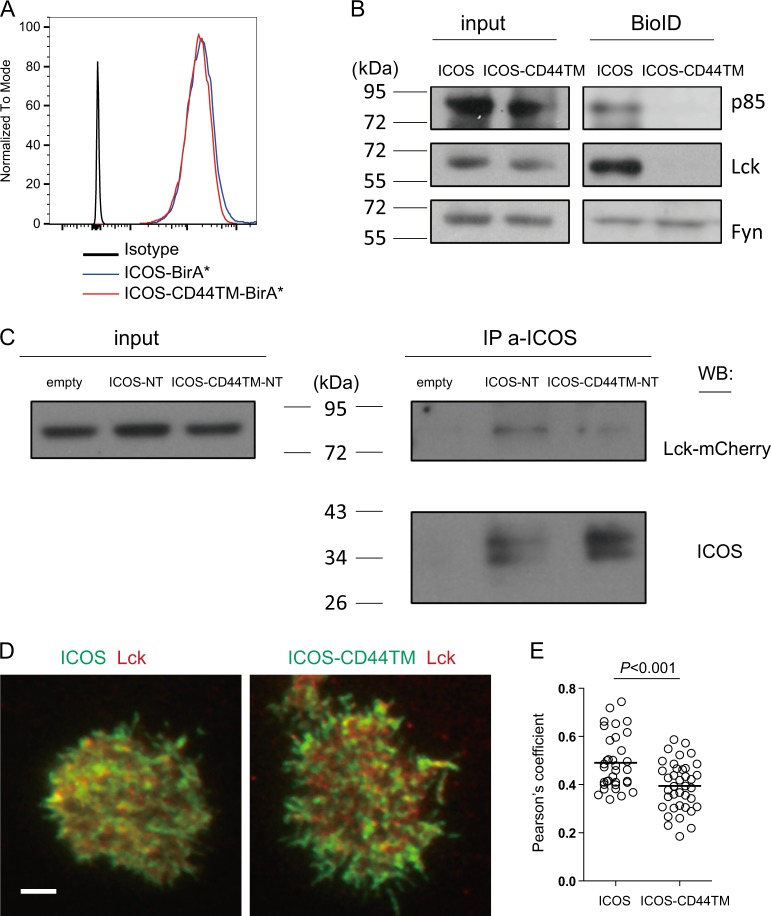
Transmembrane domain-dependent ICOS-Lck association. **a** Surface ICOS staining of Jurkat cells stably expressing BirA*-tagged ICOS or ICOS-CD44TM molecules. **b** Immunoblotting of p85, Lck, and Fyn in total lysates (input) or streptavidin-precipitated proteins from Jurkat cells that stably expressed BirA*-tagged ICOS or ICOS-CD44TM molecules after 24 h of culture in the presence of exogenous biotin. The data represent the results of four independent experiments. **c** Input (3% total lysates) or anti-ICOS immunoprecipitates of ICOS-NT or ICOS-CD44TM-NT Jurkat cells transduced with mCherry-tagged Lck were immunoblotted for p85 and ICOS. The data represent the results of three independent experiments. **d** Representative TIRF-plane images of EGFP-tagged ICOS or ICOS-CD44TM (green) and mCherry-tagged Lck (red) on Jurkat cells 10 min after being placed on anti-ICOS-incorporated planar lipid bilayers. **e** Pearson’s correlation coefficients for ICOS-Lck or ICOS-CD44TM-Lck colocalization on Jurkat cells. Each dot denotes one cell. The data presented were pooled from two independent experiments

To test whether Lck is associated with the ICOS transmembrane domain, we first resorted to the proximity-dependent biotin identification (BioID) technology that can detect interactor proteins based on transient colocalization in living cells.^20,31^ ICOS and ICOS-CD44TM molecules were linked at the C-terminus to BirA* by a relatively rigid (EAAAK)×3 linker. After overnight culture in the presence of exogenous biotin, streptavidin immunoprecipitates of Jurkat cells expressing comparable levels of ICOS-BirA* or ICOS-CD44TM-BirA* molecules were probed for p85, Lck, and Fyn (Fig. 4a). As shown in Fig. 4b, p85 was strongly labeled in the ICOS group but not in the ICOS-CD44TM group, confirming the dependence of p85 recruitment on the ICOS transmembrane domain. More importantly, Lck was now clearly detected exclusively in the ICOS group, whereas ICOS-CD44TM cells failed to yield significant Lck biotinylation. On the other hand, both ICOS-BirA* and ICOS-CD44TM-BirA* labeled a similar level of Fyn (Fig. 4b), an Src-family kinase mainly localized in the cytosol of T cells.^32^ Given that the effective labeling radius of BirA* is ~10 nm, significant numbers of ICOS and Lck molecules must be associated with one another in close proximity to be labeled. We further probed the ICOS-Lck association by coimmunoprecipitation. To this end, ICOS-NT and ICOS-CD44TM-NT Jurkat cells that expressed comparable levels of surface ICOS and intracellular mCherry-tagged Lck were incubated with an anti-ICOS antibody, washed, and then subjected to immunoprecipitation to ensure probing of ICOS-Lck association at the plasma membrane. As shown in Fig. 4c, Lck coprecipitated with ICOS-NT but not as efficiently as with ICOS-CD44TM-NT. These data demonstrate that the ICOS TMD is indeed associated with Lck under steady-state conditions.

To further examine the ICOS-Lck association after ICOS ligation, we created Jurkat cells that expressed EGFP-tagged ICOS or ICOS-CD44TM together with mCherry-tagged Lck. Ten minutes after these cells were placed on planar lipid bilayers that displayed an anti-ICOS antibody, Lck exhibited stronger colocalization with wild-type ICOS in the TIRF-imaged plane than with ICOS-CD44TM molecules (Fig. 4d, e). Together, these data strongly suggest that ICOS functions by associating with Lck in a TMD-dependent manner: Lck phosphorylates the YMFM motif to promote PI3K activation under steady-state conditions or after ICOS ligation in the bystander mode, and Lck is brought by ICOS to TCRs upon coligation to promote TCR-dependent calcium mobilization.

**Fig. 5:**
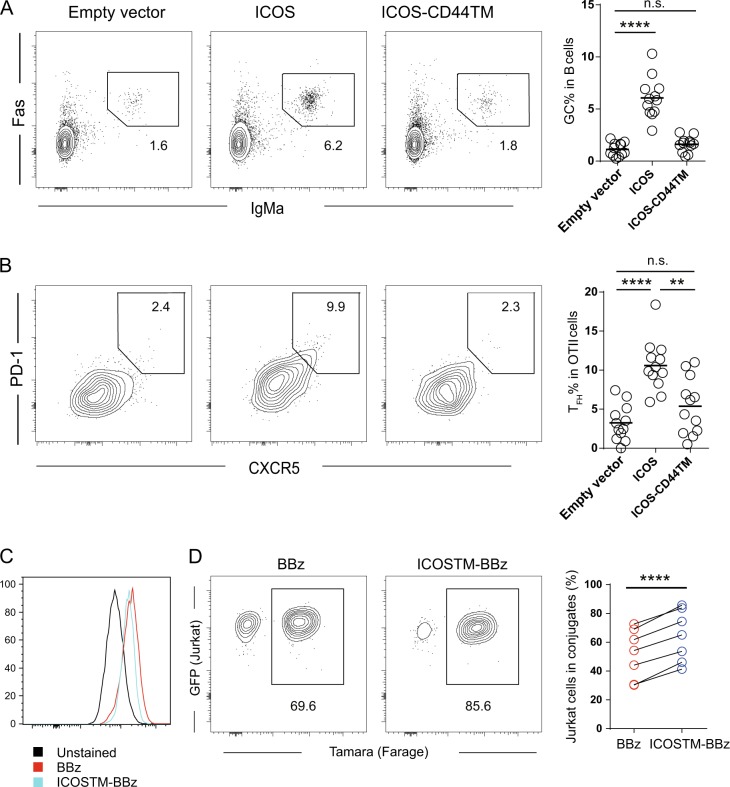
ICOS transmembrane domain-mediated helper functions. **a**, **b** Representative contour plots and frequencies of IgMa^+^Fas^hi^ GC B cells among the total CD19^+^ cells (**a**) and CXCR5^hi^PD-1^hi^ T_FH_ cells among the transferred OT-II cells (**b**). Each symbol in scatter plots represents one mouse, and the lines denote the means. The data presented were pooled from 3 independent experiments (**P < 0.01 and **** *P* < 0.0001, as determined by Mann–Whitney tests). **c** Surface CAR staining of Jurkat cells lentivirally transduced to express CD19-reactive BBz CAR, which has the original CD8 transmembrane domain, or ICOSTM-BBz CAR, which has the ICOS transmembrane domain. **d** Representative FACS profiles (left; the numbers indicate the event percentages in the gates) and summarized frequencies of CAR-expressing Jurkat cells in conjugation with TAMARA-labeled CD19^+^ Farage B cells (right). Each line connecting two dots represents one independent experiment (****P < 0.0001, as determined by paired *t*-test)

An important prediction of this model is that TMD-swapped ICOS cannot support normal GC and T_FH_ development *in vivo* even when endowed with an intact cytoplasmic domain. Indeed, when we reconstituted *Icos*^-/-^ OT-II T cells with full-length ICOS or ICOS-CD44TM and tested whether these cells could serve as competent helper T cells to promote GC formation, we found that only ICOS, not TMD-swapped ICOS-CD44TM, rendered competent helper T cells to support GC formation (Fig. 5a–c).

### Modified chimeric antigen receptors with the ICOS transmembrane domain

T cells that carry tumor-specific chimeric antigen receptors (CAR) have been successfully used to combat B-cell tumors.^33^ Much effort has been made to optimize CARs to function more efficiently, and cytoplasmic signaling motifs from various immune molecules are important building blocks in the CAR-engineering toolbox.^34^ Given the Lck-associating nature of the ICOS TMD, we replaced the CD8 transmembrane domain in the standard CD19-reactive FMC63-BBz CAR (BBz CAR) with the ICOS TMD (ICOSTM-BBz CAR) and tested the two types of receptors in Jurkat cells. As shown in Fig. 5d–f, when incubated with Farage B cells, Jurkat cells carrying ICOSTM-BBz CAR yielded more frequent T-B conjugates, indicating that the ICOS TMD can function as a signaling component independent of its native molecular context and may be useful for engineering CARs with enhanced functions.

## Discussion

A surprising insight of the current study is that the ICOS TMD signals by mediating an association with Lck. In principle, this association may involve ICOS interactions with another transmembrane proteins that can physically bind to Lck, for example, CD4, CD8, or CD28. However, the parental Jurkat cells we use do not express CD4, and CRISPR/Cas9-mediated CD28 deletion did not diminish ICOS-mediated costimulation (our unpublished data). Charged residues in the TMDs of TCR and CD3 chains help establish their intermolecular association in defined stoichiometry,^35^ although ICOS does not have any charged residues in its TMD. It is also possible that the ICOS TMD directly, or indirectly through other lipid species, interacts with the membrane-embedded myristyl and/or palmityl moieties of acylated Lck molecules. In this regard, it is interesting to note that both human and mouse ICOS TMDs contain cysteine residues, which can in principle be acylated. However, alanine replacement of these cysteines did not affect the ICOS-mediated costimulation of calcium signaling (our unpublished data). Furthermore, when alanines were used to substitute for strings of 5 amino acids in the TMD starting from the outer and going into the inner leaflet of the plasma membrane, no obvious motif could be clearly defined as being essential for calcium costimulation beyond the fact that such substitution reduced the level of surface ICOS (our unpublished data). Therefore, further tests of the abovementioned possibilities and precise elucidation of the biochemical and biophysical natures of the ICOS TMD-Lck association await improved tools for studying protein-lipid interactions and in vitro reconstitution of the signaling apparatus.

These uncertainties notwithstanding, Lck association would explain the three-tiered signaling functions that ICOS mediates for activated T cells: ligand-independent and TCR-independent p85 binding and PI3K activation under steady-state conditions, ligation-enhanced but TCR-independent PI3K activation, and enhancement of the TCR proximal signaling cascade upon coligation with the antigen receptor complex. Even without receptor ligation or TCR stimulation, ICOS can associate with Lck at a level that can be detected by both the BioID method and coimmunoprecipitation. When ICOS is crosslinked, the ICOS-Lck association most likely becomes stronger and more widespread at the cell membrane to mediate more robust p85 recruitment and PI3K activation. In combination, these observations explain how ICOS itself can apparently maintain its phosphorylated state and activate the PI3K pathway, a process that is of crucial importance for follicular T-cell recruitment and T_FH_ maintenance by follicular B cells in vivo.^10,12^ Given that the pool of Lck available to signal TCR is a limiting factor for signal initiation and propagation,^29,36^ Lck association would also mean that the ICOS receptor can bring more Lck molecules to TCR complexes that are coligated, thus explaining how ICOS can costimulate calcium mobilization triggered by low-level TCR stimulation, a process that is particularly important for antigen-specific T-B interactions in the germinal center.^15^ These findings, combined with the observation that the ICOS transmembrane domain functions independently of its native molecular context in our proof-of-principle CAR experiments, also offer a likely explanation for the recent report that third-generation CAR T cells carrying the ICOS transmembrane domain persist better and are more efficient at clearing tumor cells in NSG mice than those without the domain.^37^

## Electronic supplementary material

Figure S1

Figure S2

Figure S3

Figure S4

Figure S5
