# Supplementary material for: Transmembrane domain-mediated Lck association underlies bystander and costimulatory ICOS signaling
Source: Cell Mol Immunol. 2018 Dec 6;17(2):143–52. doi: 10.1038/s41423-018-0183-z (PMC7000777; doi:10.1038/s41423-018-0183-z)
Supplement: Supplementary file 1 — Figure S1 [file 41423_2018_183_MOESM1_ESM.pdf]

Figure S1

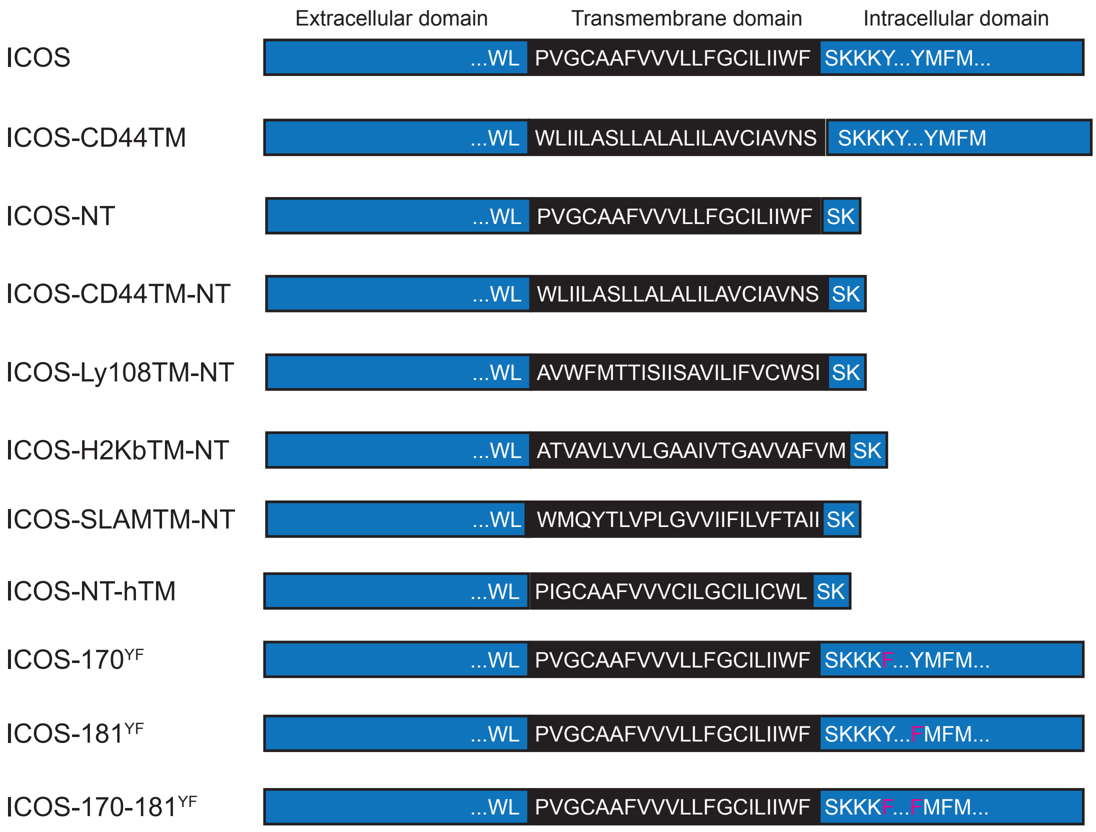
Extracellular domain
Transmembrane domain
Intracellular domain
ICOS
...WL PVGCAAFVVVLLFGCILIIWF SKKKY...YMFM...
ICOS-CD44TM
...WL WLIILASLLALALILAVCIAVNS SKKKY...YMFM
ICOS-NT
...WL PVGCAAFVVVLLFGCILIIWF SK
ICOS-CD44TM-NT
...WL WLIILASLLALALILAVCIAVNS SK
ICOS-Ly108TM-NT
...WL AVWFMTTISIISAVILIFVCWSI SK
ICOS-H2KbTM-NT
...WL ATVAVLVVLGAAIVTGAVVAFVM SK
ICOS-SLAMTM-NT
...WL WMQYTLVPLGVVIIFILVFTAII SK
ICOS-NT-hTM
...WL PIGCAAFVVVCILGCILICWL SK
ICOS-170YF
...WL PVGCAAFVVVLLFGCILIIWF SKKKF...YMFM...
ICOS-181YF
...WL PVGCAAFVVVLLFGCILIIWF SKKKY...FMFM...
ICOS-170-181YF
...WL PVGCAAFVVVLLFGCILIIWF SKKKF...FMFM...
