# Supplementary figures and images for: Transmembrane domain-mediated Lck association underlies bystander and costimulatory ICOS signaling
Source: Cell Mol Immunol. 2018 Dec 6;17(2):143–52. doi: 10.1038/s41423-018-0183-z (PMC7000777; doi:10.1038/s41423-018-0183-z)

Figure S2

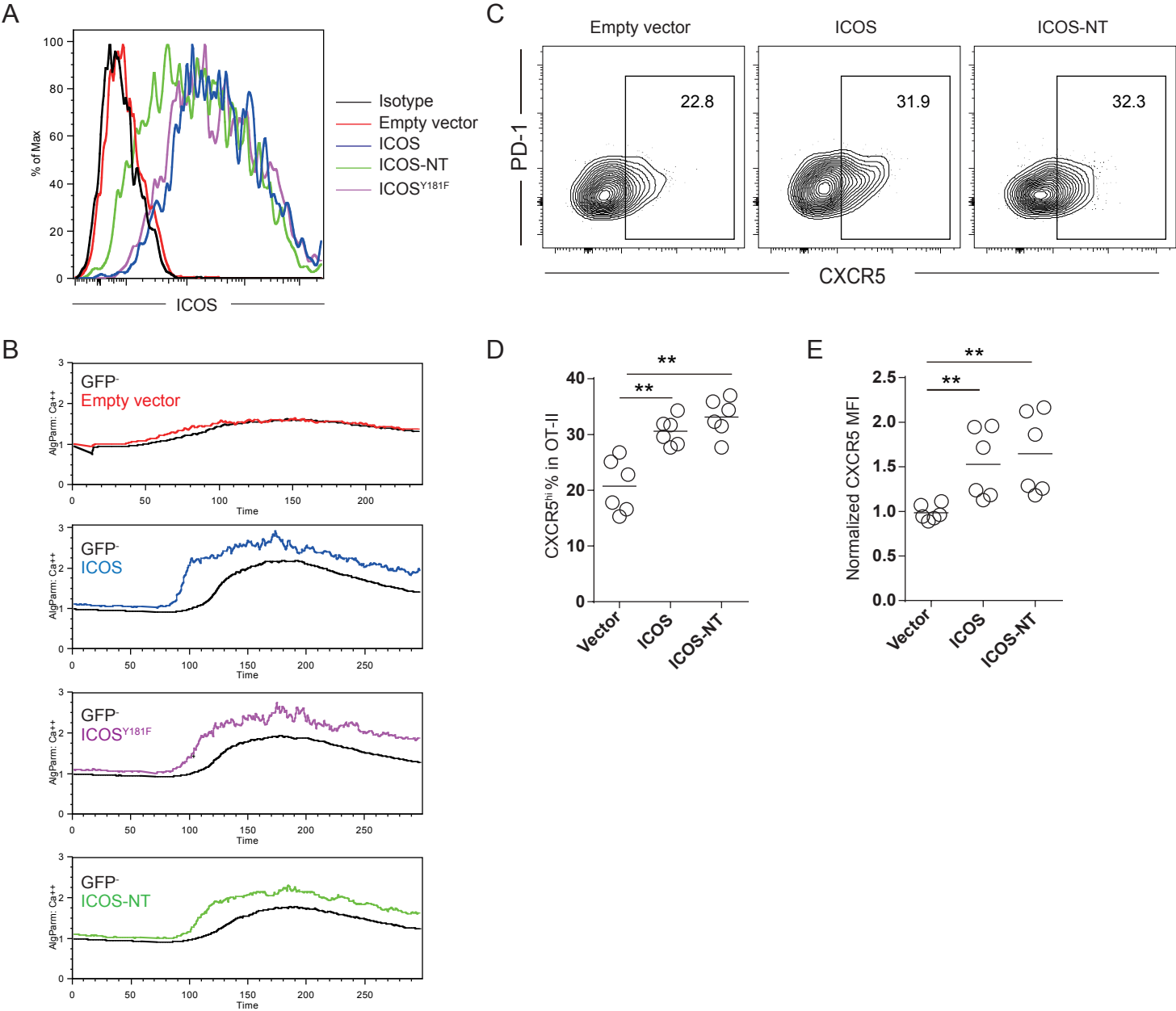

Supplement: Supplementary file 2 — Figure S2 [file 41423_2018_183_MOESM2_ESM.pdf]

Figure S3

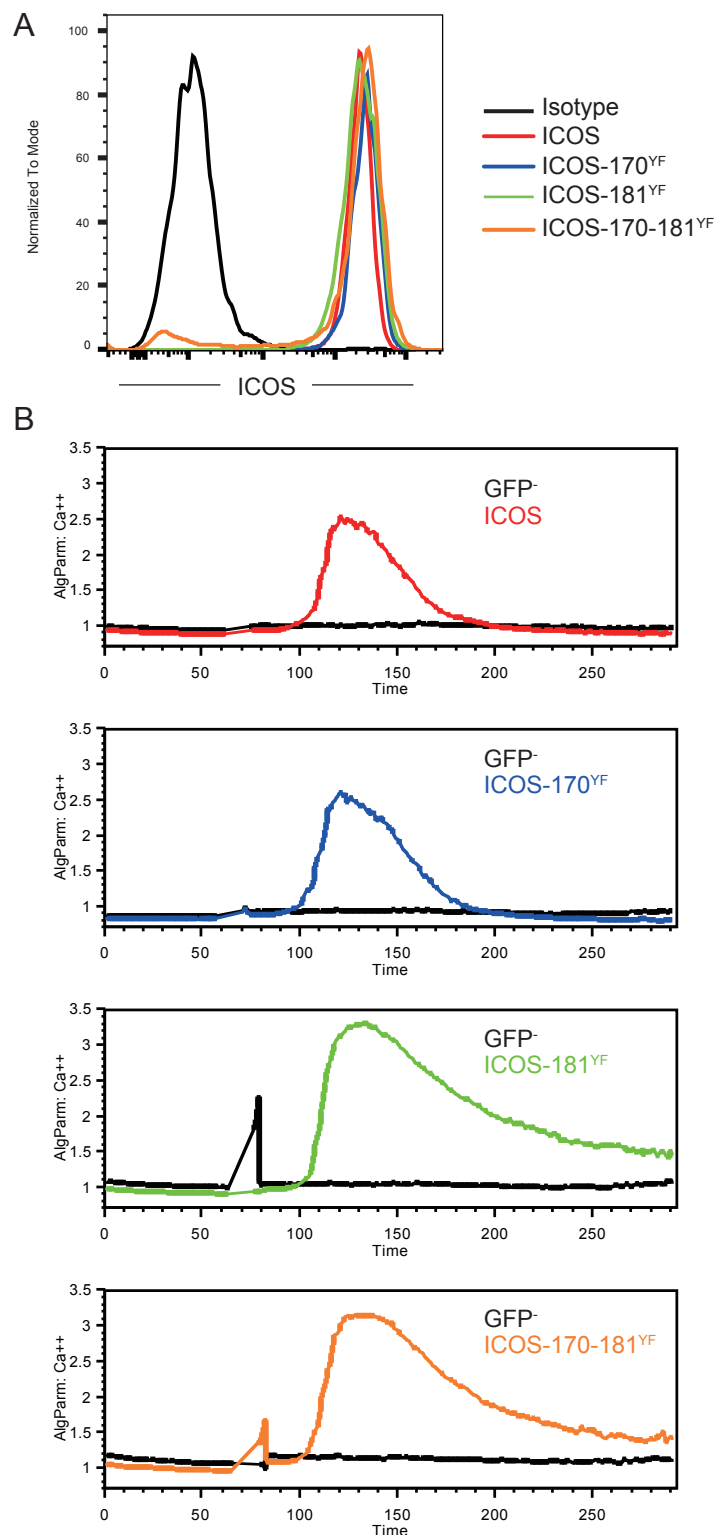

Supplement: Supplementary file 3 — Figure S3 [file 41423_2018_183_MOESM3_ESM.pdf]

Figure S4

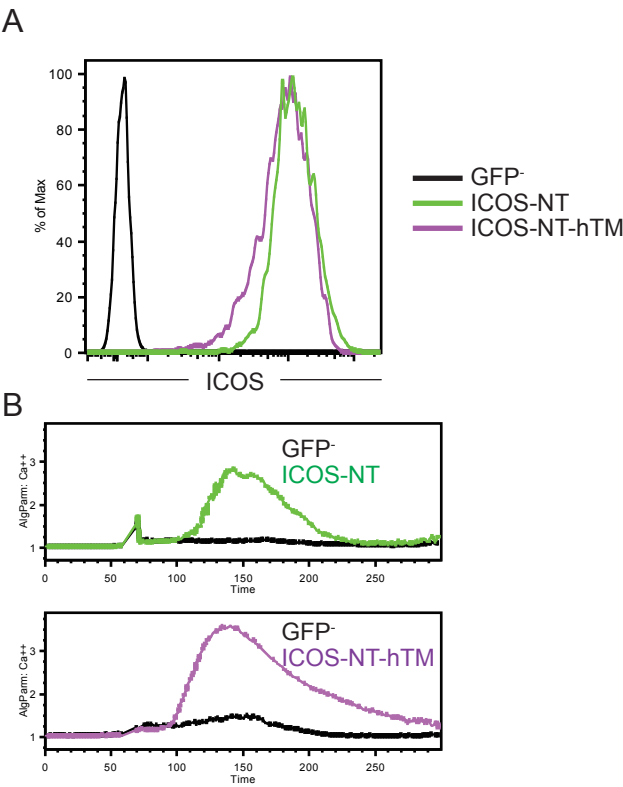

Supplement: Supplementary file 4 — Figure S4 [file 41423_2018_183_MOESM4_ESM.pdf]

Figure S5

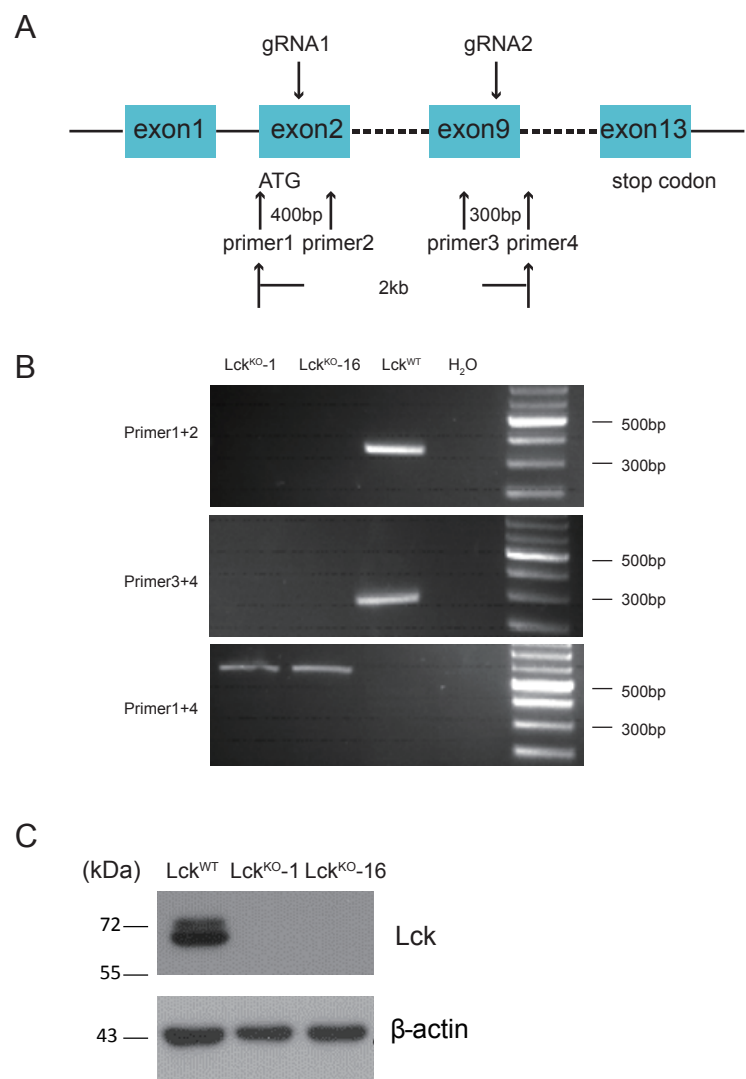

Supplement: Supplementary file 5 — Figure S5 [file 41423_2018_183_MOESM5_ESM.pdf]
